# “Why them, why me, why us?” The experiences of parents of children with lysosomal acid lipase deficiency: an interpretative phenomenological analysis study

**DOI:** 10.1186/s13023-022-02335-4

**Published:** 2022-05-12

**Authors:** S. Hassall, D. M. Smith, S. Rust, S. A. Jones, A. Wittkowski

**Affiliations:** 1 Faculty of Biology, Medicine and Health, Division of Psychology and Mental Health, School of Health Sciences, Manchester Academic Health Science Centre, The University of Manchester, 2nd Floor Zochonis Building, Brunswick Street, Manchester, M13 9NY UK; 2 Greater Manchester Mental Health NHS Foundation Trust, Prestwich, UK; 3 Manchester Academic Health Science Centre, Manchester, UK; 4 Royal Manchester Children’s Hospital, Manchester, UK

**Keywords:** Qualitative, Parents, Paediatrics, Wolman disease, Lived experience

## Abstract

**Background:**

Lysosomal acid lipase deficiency (LALD) is an ultra-rare, inherited metabolic disease within the category of lysosomal storage disorders, affecting an infant’s ability to metabolise cholesterol. Developments in treatment, including Enzyme Replacement Therapy, have proven successful, with some children living for a number of years with treatment, although the future still remains unknown. The aim of this study was to explore the lived experiences of parents of children with LALD.

**Main text:**

Participants were recruited from across the United Kingdom between 2020 and 2021. Eight parents (five mothers and three fathers) whose child had a confirmed diagnosis of LALD were interviewed. Data collected from the semi-structured interviews were audio-record, transcribed and analysed using Interpretative Phenomenological Analysis (IPA). Three superordinate and nine subordinate themes emerged from the data: (1) *Uncertainty—a double-edged sword* (plunged into an uncertain world, living life with worry and walking the tightrope of stability), (2) *Powerless against a shared battle with LALD* (a helpless parent, a joint battle, protection against distress and a vulnerable parent needing help) and 3) *Accepting a life with LALD* (coming to terms with a diagnosis of LALD and a hidden condition).

**Conclusions:**

The findings of this study highlight that the diagnosis of LALD proves to be a very challenging and emotionally distressing time in parents’ lives, with increased uncertainty about what the future will hold for their child. This study signified the importance of healthcare pathways and service provisions to support parents and their children throughout diagnosis and beyond.

## Introduction

Infantile onset lysosomal acid lipase deficiency (LALD), historically known as Wolman disease, is an ultra-rare, multi-system lysosomal storage disorder (LSD), predominantly affecting the liver, spleen and gut [1]. LALD impacts a child’s ability to metabolise cholesterol and lipids and thus causes severe vomiting and diarrhoea, an enlarged abdomen and growth failure [2, 3]. These symptoms typically present within the first six months of life and without treatment the condition progresses rapidly, proving fatal [1, 4]. Due to the rarity of the LALD, knowledge of its presentation and medical management are poorly understood, which can impact on timely diagnosis due to misunderstanding or misdiagnosis of clinical symptoms. [1, 2]

Experimental treatments over the last 30 years have been invasive with poor longer-term survival [2]. A recent multicentre international trial has found Enzyme Replacement Therapy (ERT) offered to children on a weekly basis to be effective (Clinicaltrials.gov NCT01371825 [1]. Despite the effectiveness of ERT, the complexity of LALD can result in serious complications involving frequent hospitalisations [1]. Significant digestive problems and feeding difficulties in LALD are common and can fluctuate with physical illness, thus it is not uncommon for parents to have to manage gastrostomy feeding alongside restricted diets, severe sickness and diarrhoea [1]. More recently, multimodal treatment including ERT, dietary management and Hematopoietic Stem Cell Transplant (HSCT) has been trialled in some LALD cases. Although HSCT carries significant risks, multimodal treatment offered to infants has shown an improvement in gastrointestinal symptoms in those who survived the procedure, with some children being able to eat and require less gastrostomy feeding over time [5].

As a result of the relatively new ERT treatment (EMA approved 2015), the natural progression of LALD is unknown. This lack of knowledge increases the uncertainty which parents of children with LALD face. Uncertainty within chronic paediatric conditions can result in and maintain poor mental health in parents, particularly anxiety [6, 7].

No studies to date have explored the psychological impact of LALD on parents qualitatively. Given the increased level of uncertainty and lack of knowledge surrounding LALD, it is important to understand the distinct experiences of parents at such an early stage of treatment development. The primary aim of the study was to explore the lived experiences of parents of children with LALD.

## Method

### Design

This was a qualitative study using Interpretative Phenomenological Analysis (IPA), an approach which has been used extensively to explore individuals’ experiences of physical health problems as well as parental experiences [8–11]. IPA allows for an in-depth understanding of a smaller group of homologous participants [12, 13].

All relevant ethical and research governance approvals were obtained in February 2020 by the Northwest Greater Manchester West NHS committee (19/NW/0739).

### Participant inclusion criteria

Eligible participants were identified as parents of living children with a confirmed diagnosis of infantile onset LALD. All participants were required to be proficient in English, over the age of 18 and have the capacity to give informed consent to take part.

A sample of between four and eight participants was sought which is typical for an IPA study of this scope [14, 15]. A sample of this size allows for an in-depth and detailed exploration of partcipants’ experiences [9, 16, 17].

### Procedure

The MPS Society and a specialist LALD team identified eligible participants in the UK on their mailing lists and caseloads. Parents were provided with information about the study and were required to complete a consent to contact form or contact a member of the research team to express an interest. Participants were provided with a Participant Information Sheet and instructed to contact the research team if they were interested in taking part whereby a convenient time for a telephone interview was arranged.

On the day of the interview, verbal consent was gained and participants were interviewed using a semi-structured topic guide. Consent and interviews were audio-recorded separately using an encrypted device. The topic guide was developed following discussions with a multi-disciplinary specialist LALD team and a mother of a child with LALD. A pilot interview was conducted by the principal researcher (SH) to ensure relevance and appropriateness of the topic guide, which formed part of the dataset. The interview was led by each participant’s own story and relevant prompts were used to gain a more in-depth understanding. Using a reflective style, the interviewer offered short summaries of participants’ responses to ensure interviewer understanding and offer participants the opportunity to provide more information [18]. Parents were interviewed individually even if they described experiences of the same child. This approach allowed for a holistic, in-depth understanding of different perceptions from each parent [19]. Seven parents were interviewed once, and one parent’s interview was conducted over two interview sessions.

Following the interview, participants were provided with appropriate debrief information and were offered a monetary voucher (£10) or a donation was made to a charity of their choice.

### Data analysis

Data were analysed using IPA following the steps outlined by Smith and Osborn [8, 14]. All interviews were transcribed verbatim by the principal researcher. Participants were assigned a pseudonym, and all identifiable information were omitted from the transcripts. The principal researcher listened to each interview recording multiple times and checked it against the transcript. Any reflections which came to mind during this initial process were “bracketed” [14]. In turn, each transcript was read multiple times by two members of the research team (SH and DMS). SH analysed each transcript manually using Microsoft Word by recording exploratory descriptive, linguistic and conceptual comments [14]. Subsequently, line-by-line, the transcripts and exploratory comments were transformed into emerging themes.

SH led on establishing connections between emerging themes, which were subsequently clustered into subordinate themes. Subordinate themes were grouped based on established relationships and superordinate themes were defined. Superordinate themes were checked against the interview transcript. Once this process had been completed for each transcript in turn, subordinate and superordinate themes for each participant were transferred onto paper post-it notes and laid out. Patterns and relationships between each transcript were identified and a table of superordinate and subordinate themes was developed. Themes which did not fit within the overall story of participants’ experiences were discarded. SH reviewed the table of themes across each interview transcript to ensure that it was representative of each participant’s story.

#### Trustworthiness and rigour

To ensure trustworthiness of the analysis process, the principal researcher discussed and presented their interpretation of the results to DMS. A reflective log was maintained throughout the process by SH to explore sense-making and feelings in relation to the research process, interviews and analysis.. Finally, interviews were led by each participant’s own story and the analysis process was conducted iteratively in a back-and-forth pattern returning to the raw data and thus ensuring that the analysis was driven by participants’ narratives.

### Reflexivity and positioning statement

An acknowledgement of the researchers’ reflexive position is important in qualitative research because their values and personal and professional experiences influence the research process [20]. The principal researcher adopted a critical realist position when conducting the research [21]. Cresswell and Clark interpreted this approach as “an integration of realist ontology (there is a real world that exists independently of our perception, theories and constructions) with a constructivist epistemology (our understanding of this world is inevitably a construction built from our own perspectives and standpoint)”. [22].

The academic research team (SH, AW and DMS) share an interest in understanding and supporting the psychological wellbeing of parents of children. SH was a practicing trainee clinical psychologist with several years’ experience working with children and families affected by rare metabolic conditions. DMS was an experienced researcher within health psychology, and AW was a clinical psychologist and experienced researcher, with a particular focus on maternal mental health and parenting. Both DMS and AW were mothers and, whilst not a parent, SH was working within an early attachment service. Co-authors SR and SJ were experts working within the field of metabolic medicine, SR approaching the research from a clinical neuropsychological background, and SJ from a medical perspective.

## Results

### Sample

The first eight eligible participants who were provided with information about the study from their medical team or MPS Society took part in the study. One parent who expressed an interest in the study did not meet the eligibility criteria. Of the eight participants, both mother and father of three children and two mothers of an additional two children took part in the study. Five parents identified themselves as primary caregivers for their child (three mothers and two fathers), all children were below the age of 10. The population of children in the sample included those who have undergone HSCT and remain on ERT and substrate reduction (dietary treatment), children who have undergone HSCT and are no longer receiving ERT or substrate reduction and untransplanted children treated with ERT and substrate reduction. Further demographic details have not been disclosed to maintain participant confidentiality.

Interviews lasted an average of 66 min (standard deviation of 15 min) and took place from September 2020-February 2021.

### Findings

**Fig. 1:**
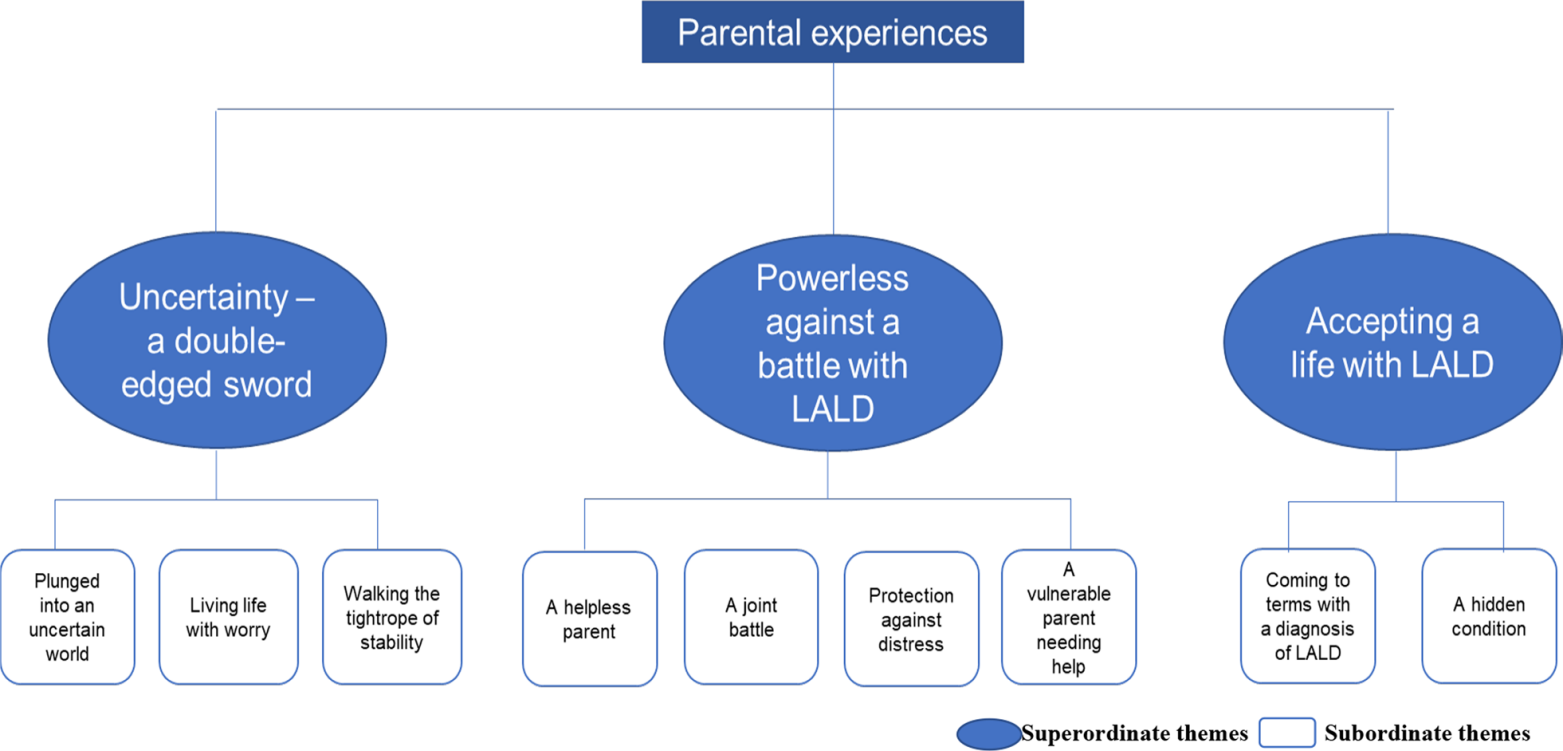
Superordinate and subordinate themes which emerged from parents’ experiences  superordinate themes,  subordinate themes

Three superordinate themes and nine subordinate themes were established (see Fig. 1). All superordinate themes represented all participants’ stories, recognising the journey and process which parents go through when receiving diagnosis of LALD which is surrounded by uncertainty. The themes capture parental vulnerabilities and the importance of regaining a sense of a normal life. Quotes will be reported to give meaning to each theme and have been carefully selected to maintain anonymity of participants.

#### Superordinate theme 1: uncertainty—the double-edged sword

In their interviews, parents explored the level of uncertainty that surrounded the diagnosis and treatment of LALD. They reflected on how it felt not having many other children with LALD who they could compare their child against which impacted on how they were able to make sense of the diagnosis and consider a future for their family. Theme 1 comprised three subordinate themes and reflected the parents’ struggles with an uncertain and conflicting, expectation-changing situation.

##### Subordinate theme 1.1: plunged into an uncertain world

All parents spoke about the uncertain world which they were hastily immersed into when their child got sick, mirroring the rapid deterioration of their child’s condition. Parents reflected on the diagnosis of an incurable and rare condition as being unexpected and extremely challenging. The little to no knowledge that they had about LALD made it difficult for parents to contemplate and make sense of the diagnosis and what that meant for them and their child given the historically high mortality rate and few living children with the condition. This uncertainty was unbearable for parents and increased distress:

That was the worst part of it, I found that you’re constantly living in fear, I was treating each day like my last day, I felt like I had to make memories every day, I felt like I had to take so many photographs ‘cause it could be my last day (Anna).

The lack of information available to parents at the point of diagnosis paralleled the lack of control that they felt as a result. Parents felt “lost” (Andrew) and this feeling led to parents desperately in search of some answers. Anna described “holding onto” the doctor’s words, whereas other parents were relying on the internet to find devastating information:

So, we googled it and the research that we did behind that said that (child) weren’t going to survive over 6 months (Hassan).

Parents described different ways of coping with not having any answers about what exactly the condition was, if there was a treatment and whether their child was going to survive during the initial period of diagnosis. For some parents, their child was so sick that they were unable to think about anything other than how their child was at the time: “You know, you live for the moment, you live for the minute, until (child) gets better” (Anna).

Patrick, however, found himself consumed by thoughts about his child’s future:

…you were going to be wondering to a certain degree is (child) going to be ok, feed ok, talk, do you know, all this stuff…and it was just multiplied by 1000, like, with me, you know every kind of aspect of (child)’s life, (child)’s future…and then the transplant, and even down to (child) won’t be able to have (child) own family because you know, the transplant, the chemo, every kind of thing was just, I suppose it was just crushing me with thoughts, you know (Patrick).

Hassan shared this way of thinking to some degree, wondering when his child was going to be “perfectly fine”: “We were thinking when is (child) going to get better, is (child) going to eat, when is (child) going to come off (child)’s feed, or can he have (child)’s life back to normal.” For Hassan, it was unbearable for him to see his child so vulnerable and sick, maintaining hope by imagining a future:

I think to myself if (child)’s not going to eat… maybe in a year’s time, 18 months times (child)’s going to be able to eat completely, and stuff like that (Hassan).

##### Subordinate theme 1.2: living life with worry

For some parents tolerating the uncertainties of the future proved a huge challenge. Maryam felt that the uncertainty left her feeling anxious and scared about what could happen in the future:

…But when they’ve got such a rare condition where they haven’t got information, you’re always in that worry… you’re thinking ‘what will happen, what will happen’” (Maryam).

Anna explained that she found it difficult not knowing how LALD was affecting her child internally, which maintained her sense of fear:

…You get these impulses you know that you’re just paralysed with fear like, you just don’t know what’s going to happen… it would knock you over when it hits you like. You know, (child) is sick… what does the future hold, what is going to happen, you know. And when that happens, like, it is really scary (Anna).

For a number of parents this worry seemed to manifest in needing to remain close to their sick child which was seemingly driven by the fear of something happening if they were not there. For Aleena, being away from her sick child was unbearable and resulted in distressing dreams that her child was crying out for her. Whilst this had improved for some parents as their child’s condition had improved, others still found this difficult, as Neha explains:

I’ll constantly be ringing throughout the day because I want to make sure (child)’s ok. It’s a constant cycle. It never stops.

Similarly, although parents talked about their worry reducing as their child’s condition stabilised, parents described an underlying constant worry:

“Granted we have the treatment and we have more reassurance but we are still living in fear. Not as much, but we are still living in fear” (Anna).

##### Subordinate theme 1.3: walking the tightrope of stability

As their children began to regain stability for longer periods, some parents could shift their focus on to how well their child was at the present moment as a means of coping with the fear of the unknown, Aleena simply said “No, I don’t think about the future, no. I just think about now and how (child) is now”.

For other parents, wellness seemed to support acceptance of uncertainty and the lack of answers. Although not having the answers was difficult for Andrew at first, not knowing what the future held allowed him to focus on the present:

… this is going very well for (child) now, everyone is very happy with (child) now and that’s where we’re at and we’re happy with it..

Hassan’s experience was similar to Andrew’s; however, Hassan preferred to focus on the improvements that his child had made: “Because I don’t want to think back to that hard time, it was tough for us, it was tough for (child). But I don’t want to think about that… I want to think about the future now.”

However, parents’ experiences demonstrated the fragility of the child’s wellness and the associated risks of LALD. Complications of LALD can often come without warning, putting parents “life on hold” (Neha) once again. This quickly transports parents back unexpectedly to the uncertain world and past experiences which perhaps have not been processed:

But when (child) does then say get sick and then you’re back in that environment again and (child)’s going through everything again, it does bring all those memories back to us (Anna).

#### Superordinate theme 2: powerless against a shared battle with LALD

This theme captured the psychological symptoms that parenting a child with LALD can elicit from feeling like a helpless counterpart within a battle against the condition which they were fighting alongside their child. It provided a deeper understanding of how the role of the parent was navigated throughout this battle. Parental vulnerabilities were encapsulated, understanding how they developed ways to protect both their child and them, which was explored within four subordinate themes.

##### Subordinate theme 2.1: a helpless parent

The parental role changed when their child was diagnosed with LALD, leaving parents feeling a sense of helplessness and powerlessness—unable to care for their child in that way that they once were. This experience occurred for parents at different times, such as at the point of diagnosis and when their child had to undergo medical procedures or treatment. Cara found the hospital environment challenging in relation to navigating her role as a mother, going through a process of claiming her baby and resenting the fact that other people were providing care for her child. She fought against the condition in this respect, reclaiming her role to support her own unmet mothering needs:

…I insisted on weaning (child) myself in the hospital, going shopping, getting all these bits of babies food and things, but it was my instinct I suppose, and nine times out of ten it wasn’t working… just to keep up that thing that I could do… but again, I think it was just to be in use (Cara).

For fathers, their acquired role of a protector of their child was threatened, Andrew described relinquishing this role to the doctors and medicine as there was nothing which he could do:

I think it took away from you…it took it away as in… but I felt that…I knew I couldn’t protect (child)… but I know when (child) was born and (child) was slipping away, the doctors in (place) and the doctors in (place) they gave us (child) back.

This feeling was shared by Patrick who wished for there to be more that he could do, yet had to succumb to not being able to do anything. This extended to finding it more difficult to attune to his child’s needs, with the added complexity of LALD:

We were helpless really, but … Yeah, helpless, I suppose. You couldn’t do anything for (child) really (…)… but like then when they’re that small you just don’t know… everything was just guessing and trying our best.

##### Subordinate theme 2.2: a joint battle

The language which parents used conveyed a “fight” against LALD which was fought by both parent and child. The dialect which parents used to describe this fight depicted a joint battle, as if parents were physically taking on the condition for their child: “I was watching their condition and my condition” (Maryam).

The battle which parents faced was embroiled with loss—from the imagined loss of their child, the loss of a healthy baby, and the cumulative losses associated with living within a hospital environment for a significant period, such as loss of their support network and temporary or permanent loss of employment. Thus, there was a felt sense of grief which parents explored in different ways, such as through anger, disbelief, and extreme sadness:

…we didn’t know… you know… how long we would have (child) and that was… both of us were afraid of that (Andrew).

…Not able to see your friends, see your family…and staying in hospital always… and it used to bring you down. You have no social life; you were just with the poorly kid there (Maryam).

For some parents, the losses associated with LALD felt more permanent, having a lasting impact on parents’ sense of who they were as a person and their mental health—often feeling isolated from the life they once had:

… I’ve become more socially awkward, I found it difficult doing normal things, seeing normal people. You just kind of become a person that lives within the four walls of the hospital or the home. You’re not you anymore (Neha).

##### Subordinate theme 2.3: protection against distress

Parents spoke openly about the psychological distress they experienced with language that described the sheer strength of their emotions: “terror”, “fear”, “stress”, “depression”, “anxiety” and “trauma”. This was conveyed by parents in relation to different aspects of LALD (diagnosis, long-term or frequent hospitalisation, treatment and the relentlessness of care related to digestive symptoms) and parenting a child with LALD more generally. During the initial period of severe illness, parents particularly described such difficult emotions as incapacitating and so often pushed emotions aside to protect themselves, their child, and their role as a parent:

I think I just blocked everything out and just (…) I mean I just wanted to curl up in a ball and forget about it (…) So, you have to get on with it and that’s it (…) Isuppose I went into survival mode (Patrick).

As time went on, the child’s wellness became protective for parents, which for some helped to manage difficult emotions associated with LALD and earlier experiences. Stability in the child’s condition led to less frequent hospitalisations – something which was significant for a number of parents. This was also supported by parents’ confidence and acquired knowledge in managing certain aspects of the condition:

… you just dread going in. And if it is just a cold and you go in, you’re on your own and there’s no getting a break and stuff… so that makes it more difficult. But I think as time has gone on now, d’you know and you get to know (child) more you can make the call if it’s just a cold or a cough or something else, do you know” (Patrick).

Despite this, attempts to block out intolerable emotions and the protection that the child’s wellness offered parents were sometimes limited in their ability to shield parents from the raw, strong, and perhaps unprocessed emotions that they harboured:

It was a traumatic experience, I still have, you know, I often get flashback memories (…) And whatever obviously I get a flood of emotion and I deal with it (Cara).

##### Subordinate theme 2.4: a vulnerable parent needing help

The diagnosis of LALD was an extremely distressing time for parents and recalled in great detail. It was felt by a number of parents that the way that the diagnosis was given to them by a professional who did not know about the condition was lacking the containment that they needed, being left to face it on their own. In addition to the psychological consequences felt by parents after receiving the diagnosis of LALD, the loss of the safety of their own home due to prolonged hospitalisation and their support network, parents were in the midst of an already vulnerable time as a new parent, as Cara pointed out:

Wolman’s has generally always been diagnosed in the early infancy so that’s a very vulnerable time, well it was for … you have all the postnatal stuff that would go with that as well and not feeling yourself as well, as well as dealing with big traumatic event, it’s really hard.

Parent’s own vulnerability echoed that of their young infants. They needed to be contained through this time and metaphorically held by the people around them in order to have the capacity to then be there for their child. For Andrew and Patrick, they felt that it was their role as a husband to hold their wives through this challenging time:

But I also feel that I want to protect (wife). You know and …like…erm the questions that (wife) would have, I would just try to reassure her…. You know questions like ‘how is this going to end’… you know… do I think that (child) is improving, do I think (child)’s looking a bit better… and I would just try to reassure her. I think I had an added pressure as a husband because of that (Andrew).

Patrick did this by remaining the positive figure, providing his wife with the space which she needed to cope, surrendering his own needs at this time:

I suppose I looked at… I suppose all day everyday nearly my wife was crying; she was very down and very upset so I suppose we both couldn’t be like it… that was the way I looked at it… one of us needed to be somewhere positive (Patrick).

It was clear that Andrew and Patrick needed holding too, with Patrick turning to family and Andrew finding this from the staff in the hospital and showing that he too needed to be looked after. This was shared by other parents too – the relationships that they had with staff proving paramount, with Hassan describing them as “family”. The continued relationship with the professionals had also been key for Anna in providing her with the containing reassurance that she needed:

You know, you’re hanging on every word they say, and like, our doctor… willjust say to us, (child)’s doing fantastic now, (child)’s doing really well now, (child)’s beaten all the odds. (child)’s really good at drilling that into us (Anna).

As well as their faith in religion, meeting other families also played a pertinent role in containing and holding parents through this time. This was particularly heard in the experiences of Neha, Aleena and Maryam. On finding the strength to get through such a difficult time for her, Neha said:

Honestly, my faith in god. My faith in god and meeting that lovely parent in the hospital, she really helped me though it all. Any time I’d ring her in the day she’s there to talk to – she’s brilliant (Neha).

#### Superordinate theme 3: accepting a life with LALD

This theme explored how parents came to terms with a life with LALD and the circumstances which were important in allowing them to be able to move towards acceptance of the condition being part of their lives. The two subthemes, *trying to make sense of ‘why?*’ and *a condition disguised by normality,* are closely linked, firstly exploring how parents were able to make sense of the question “why us” and what had helped to support their acceptance of the condition.

##### Subordinate theme 3.1: coming to terms with a diagnosis of LALD

Parents found different ways to make sense of the diagnosis. Some opted to focus on the positives and religious aspects of them being “chosen” to be parents of a child with LALD, finding solace in the fact that they could provide the best possible care that the child needed:

…don’t ask ourselves a negative of why us, I said ask ourselves a positive thing, that of all the parents in the whole world that could have a child with Wolmans, we were chosen to have (child). Because god knew that me and (wife) were going to be the best parents in the world for (child) (Andrew).

Other parents, however, were entangled with blame. Cara described blame intertwined with anger which was projected onto the staff looking after her child. For Patrick and Neha, however, this sense of blame was projected inwardly at themselves. Patrick felt as though this was a punishment for living a pleasant life; however, for Neha there seemed to be an internalised critic from a misinformed outsider perspective:

…it was just too much to take in especially as some people say it’s your fault, you’ve done something wrong, that’s why your child is like that... There was just so many things and it was like, it was too much. Too much to take in.

##### Subordinate theme 3.2: a hidden condition

Diagnosis of LALD was life-changing and difficult for parents to come to terms with, with some parents describing how their life revolved around the condition. For some parents, acceptance of the condition was seemingly forced by a lack of choice rather than a process. Despite this, it was clear that it became easier to accept the diagnosis as their child’s condition improved, allowing some parents to shift from the image of a poorly baby to an able child. Talking about acceptance of the diagnosis, Cara said:

It’s (child’s) wellness, I know it is. (child)’s erm developed and (child)’s well and that. A huge thing for me was around (child)’s cognitive ability I suppose, I don’t know. I was just kind of really concerned. It’s one thing to have a physical health problem but then to have …. You know to be cognitively impaired as well. So that was always a worry.

Children meeting milestones was significant for parents, seemingly attached to the anticipated loss of their child before they reached such a point. This was exemplified by the elation described by some parents as a result of their child’s first day of school. It was clear from all parental accounts that the milestone of their child beginning to eat was particularly important for them to achieve, which was often accompanied by a reduction in vomiting and diarrhoea which had previously been constant. Eating (and a reduction in digestive symptoms) was intertwined with the child (and parents) being able to live a normal, unrestricted and fulfilling life:

…a lot of them don’t eat, erm and are peg fed and have very limited diets, so that was always… that would be a big worry for me… and my wife… and it would just make life more difficult again… if (child) he was peg fed… it would limit him in a lot of ways for what (child) could do and couldn’t do. I just wanted (child) to have the choice of anything (Patrick).

Despite children having a restricted diet, most parents viewed the diet as “relatively normal” and a manageable aspect of the condition which they felt in control of. Anna described dietary management as “our normal”. For other parents like Aleena and Maryam, however, the fact that their child was unable to eat a *normal* diet alongside their family was more challenging—setting their child aside as *different*, something which some parents could not, or did not want to, contemplate:

…but now I’m not thinking about it… now I’m just think (child) is well and treat (child) like normal kid… sometimes in my mind I just think (child) is healthy and everything (Aleena).

The fact that their children appeared visually “normal” with nobody being able to tell that their child had LALD was a point which was raised by parents throughout, again enabling acceptance. The condition being disguised in a “normal” body protects parents from constantly being confronted by the diagnosis and the emotions which are attached to it:

…So, if you were to explain to someone what it was, they wouldn’t be able to tell because (child) looks completely normal… (Hassan).

Parents spoke about the significant disruption of ERT initially being given to their child in a hospital setting. Although not without some challenges, the transition to treatment at home also facilitated parents and their children living an unrestricted life, allowing them to rebuild a version of the life that they had left behind:

… So, six hours every Monday…travel to (place) and back (…) so you were in hospital from, you were on the ward for half four in the morning and you were lucky if you were home for eight in the evening. So, erm…to get home treatment is like, another world to us (Anna).

## Discussion

This study explored the lived experiences of parents with a child with LALD. Parents faced significant psychological challenges upon receiving the diagnosis of such a rare condition with an uncertain prognosis and long hospital stays. Parents expressed considerable vulnerability and distress. Although an underlying worry which could sometimes become overwhelming remained with parents, the child’s wellness because of treatment considerably supported parents’ ability to come to terms with LALD.

Parents recalled the point of diagnosis in great detail. Diagnoses were often delivered by a professional with no knowledge of LALD and sometimes the mode of delivery lacked compassion, increasing distress. This way of receiving a diagnosis is not uncommon for LSDs [23]. In their meta-synthesis, Kepreotes, Keatinge and Stone found that sadness and chronic grief were common in parents of children with physical health conditions, with the time of diagnosis being parents’ most vulnerable time [24].

The uncertainty surrounding LALD was difficult for parents to bear upon receiving the diagnosis with information about LALD being largely unavailable. Parental adjustment of rare genetic conditions has been found to be assisted by knowledge surrounding the condition, which in turn facilitated parents to comprehend the diagnosis [25]. Although uncertainty remained a huge challenge for some, a novel finding of this study demonstrated a psychologically protective capacity of uncertainty after the period of acute illness for some parents compared to other LSDs, allowing them to focus on the present moment [23].

Guilt and self-blame can be a common experience for parents of children with chronic conditions, particularly those which are genetic. Whilst this was an anticipated finding, it was not something which was reported by parents in the current study. However, self-blame and guilt were understood by a means of living a nice life. Perhaps this finding highlighted differences between X-linked genetic conditions in which feelings of guilt and self-blame have been found to be increased compared to autosomal recessive conditions such as LALD [26].

### Clinical and research implications

This study encapsulated the psychological experiences and needs of parents which need to be considered within service structures and specialist psychological provision for children with LALD/LSDs. Differences in the way that mothers and fathers acutely managed distress was pertinent in the study, with mothers able to express their distress more overtly, whereas fathers assumed a role of strength for both their partner and their child. Although previous research has demonstrated a tendency for mothers to assume primary caregiving responsibility, this study captured the voices of fathers who assumed this role. Future research exploring the differences between mothers and fathers as primary caregivers for children with chronic physical health conditions would be an interesting development within the field of metabolics. This knowledge would allow services to support the idiosyncratic psychological care needs of parents within paediatric services. Increased psychological support for parents has been linked to improved adjustment and better physical and emotional health outcomes [27, 28].

Parents have significant psychological needs when their child receives a diagnosis of a chronic condition which needs to be addressed within protocols of delivering a diagnosis. Further research using a trans-diagnostic sample of parents would be helpful in determining better ways for professionals to deliver diagnoses to alleviate ongoing distress.

Parental feelings towards a modified diet were varied, highlighting important cultural considerations which were beyond the scope of this study. Future research will be important in understanding cultural differences in chronic paediatric health conditions which require adherence to dietary management to support the child’s wellness.

### Strengths and limitations

This study was the first of its kind to explore the lived parental experiences of children with LALD, which using IPA allowed for. A real strength of this study was the inclusion of fathers’ stories enabled by individual interviews. The use of multi-family member interviews allowed for an in-depth holistic view and multiple perspectives to answer the research question [19]. It should be recognised that this study was conducted across the UK and therefore results might not be transferable to the different healthcare systems which could impact parents’ experiences.

## Conclusion

This study explored the in-depth lived experiences of parents whose child had a diagnosis of LALD. The study highlighted the significant impact that a diagnosis of LALD has on parents, uncovering their psychological experiences from the point of diagnosis to living with a high degree of uncertainty. The study explored how parents have been able to come to terms with the diagnosis, facilitated by their child being able to live an unrestricted life as a consequence of treatment giving families hope. The psychological vulnerability of parents at the time of diagnosis, living within hospital and deterioration in their child’s condition needs to be considered and addressed within service provision to provide specialist psychological support for parents.

## Data Availability

The datasets generated and/or analysed during the current study are not publicly available due to maintaining participant confidentiality and anonymity but are available from the corresponding author on reasonable request. Ethics approval and consent to participate All relevant ethical and research governance approvals were obtained in February 2020 by the North West Greater Manchester West NHS committee (19/NW/0739).

## Abbreviations

ERT: Enzyme replacement therapy
LALD: Lysosomal acid lipase deficiency
HSCT: Hematopoietic stem cell transplant
IPA: Interpretative phenomenological analysis
